# Supplementary material for: National spatiotemporal patterns of influenza-like illness in Iran: A pre-COVID-19 study (2015–2019)
Source: PLoS One. 2025 Apr 21;20(4):e0320990. doi: 10.1371/journal.pone.0320990 (PMC12011232; doi:10.1371/journal.pone.0320990)
Supplement: S1 Table — (DOCX) [file pone.0320990.s001.docx]

| **Data Source** | **Description** | **Time Period** | **Data Coverage** | **Exclusions/Adjustments** |
| --- | --- | --- | --- | --- |
| Iranian Influenza Surveillance System (IISS) | Retrospective ILI data | 21 March 2015 - 22 March 2019 | 109,919 ILI patients registered | 752 cases excluded due to lack of residency addresses |
| Iran’s Population and Housing Census | Census population data | 2016 | Population data for all counties in Iran (78,154,662 people) | _ |
