# Supplementary material for: National spatiotemporal patterns of influenza-like illness in Iran: A pre-COVID-19 study (2015–2019)
Source: PLoS One. 2025 Apr 21;20(4):e0320990. doi: 10.1371/journal.pone.0320990 (PMC12011232; doi:10.1371/journal.pone.0320990)
Supplement: S2 Table — (DOCX) [file pone.0320990.s002.docx]

| **Month** | **ID** | **Month** | **ID** | **Month** | **ID** | **Month** | **ID** |
| --- | --- | --- | --- | --- | --- | --- | --- |
| 21 March 2015 - 20 April 2015 | 1 | 20 March 2016 - 19 April 2016 | 13 | 21 March 2017 - 20 April 2017 | 25 | 21 March 2018 - 20 April 2018 | 37 |
| 21 April 2015 - 21 May 2015 | 2 | 20 April 2016 - 20 May 2016 | 14 | 21 April 2017 - 21 May 2017 | 26 | 21 April 2018 - 21 May 2018 | 38 |
| 22 May 2015 - 21 June 2015 | 3 | 21 May 2016 - 20 June 2016 | 15 | 22 May 2017 - 21 June 2017 | 27 | 22 May 2018 - 21 June 2018 | 39 |
| 22 June 2015 - 22 July 2015 | 4 | 21 June 2016 - 21 July 2016 | 16 | 22 June 2017 - 22 July 2017 | 28 | 22 June 2018 - 22 July 2018 | 40 |
| 23 July 2015 - 22 August 2015 | 5 | 22 July 2016 - 21 August 2016 | 17 | 23 July 2017 - 22 August 2017 | 29 | 23 July 2018 - 22 August 2018 | 41 |
| 23 August 2015 - 22 September 2015 | 6 | 22 August 2016 - 21 September 2016 | 18 | 23 August 2017 - 22 September 2017 | 30 | 23 August 2018 - 22 September 2018 | 42 |
| 23 September 2015 - 22 October 2015 | 7 | 22 September 2016 - 21 October 2016 | 19 | 23 September 2017 - 22 October 2017 | 31 | 23 September 2018 - 22 October 2018 | 43 |
| 23 October 2015 - 21 November 2015 | 8 | 22 October 2016 - 20 November 2016 | 20 | 23 October 2017 - 21 November 2017 | 32 | 23 October 2018 - 21 November 2018 | 44 |
| 22 November 2015 - 21 December 2015 | 9 | 21 November 2016 - 20 December 2016 | 21 | 22 November 2017 - 21 December 2017 | 33 | 22 November 2018 - 21 December 2018 | 45 |
| 22 December 2015 - 20 January 2016 | 10 | 21 December 2016 - 19 January 2017 | 22 | 22 December 2017 - 20 January 2018 | 34 | 22 December 2018 - 20 January 2019 | 46 |
| 21 January 2016 - 19 February 2016 | 11 | 20 January 2017 - 18 February 2017 | 23 | 21 January 2018 - 19 February 2018 | 35 | 21 January 2019 - 19 February 2019 | 47 |
| 20 February 2016 - 19 March 2016 | 12 | 19 February 2017 - 20 March 2017 | 24 | 20 February 2018 - 20 March 2018 | 36 | 20 February 2019 - 20 March 2019 | 48 |
