# Supplementary figures and images for: National spatiotemporal patterns of influenza-like illness in Iran: A pre-COVID-19 study (2015–2019)
Source: PLoS One. 2025 Apr 21;20(4):e0320990. doi: 10.1371/journal.pone.0320990 (PMC12011232; doi:10.1371/journal.pone.0320990)

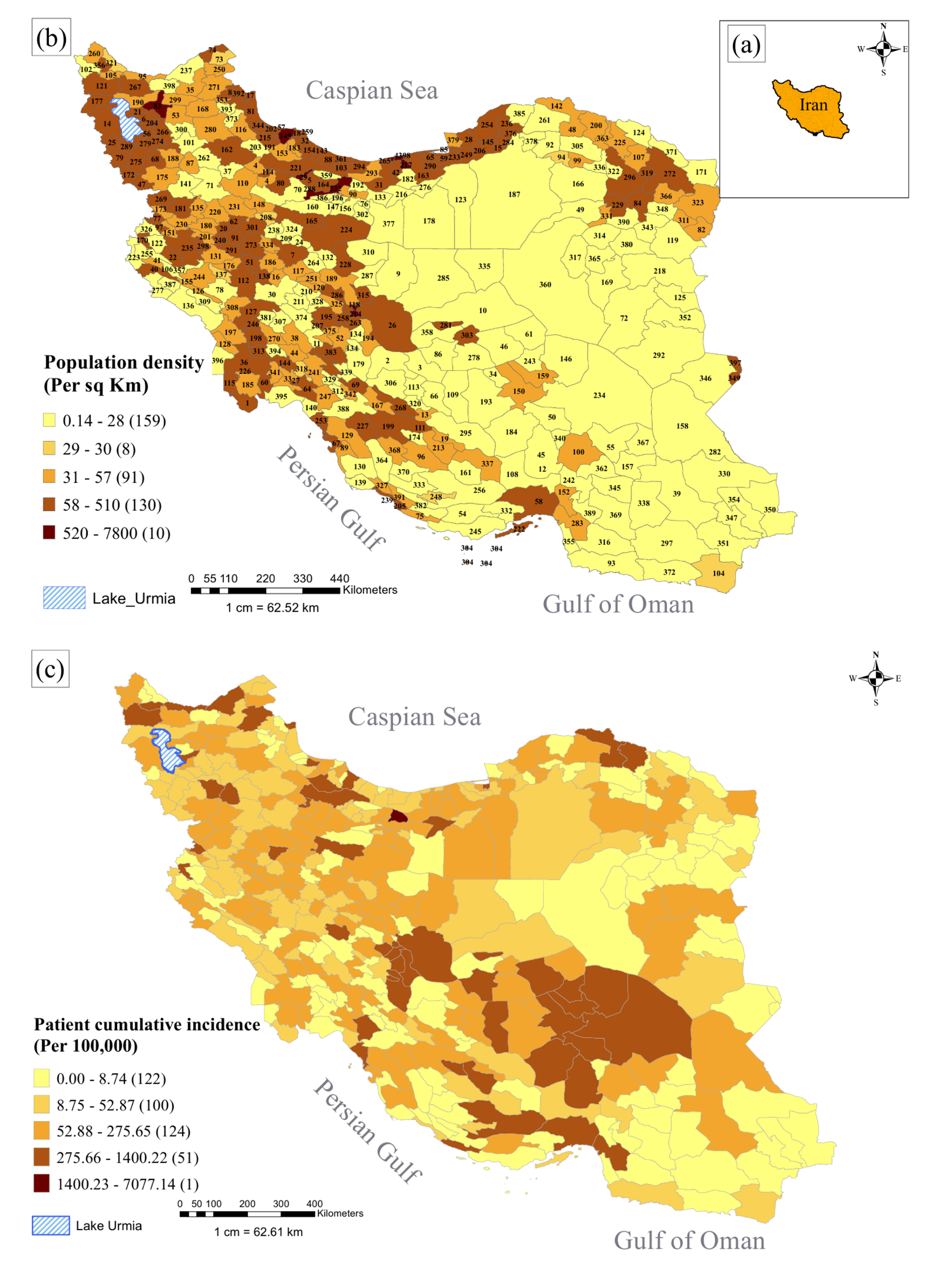

Supplement: S1 Fig — (b) Iran’s population density per square kilometers (Geometrical Interval)- The colors ranging from pale yellow to dark brown indicate the population density of different counties, based on the 2016 census data. (c) The map shows the 398 counties of Iran (Geometrical Interval), with colors ranging from pale yellow to dark brown indicating the Influenza like Illness incidence rate from March 2015 to March 2019. The identification codes for the study locations (counties) are presented in each polygon. The maps have been created using ArcGIS software. (TIF) [file pone.0320990.s003.tif]

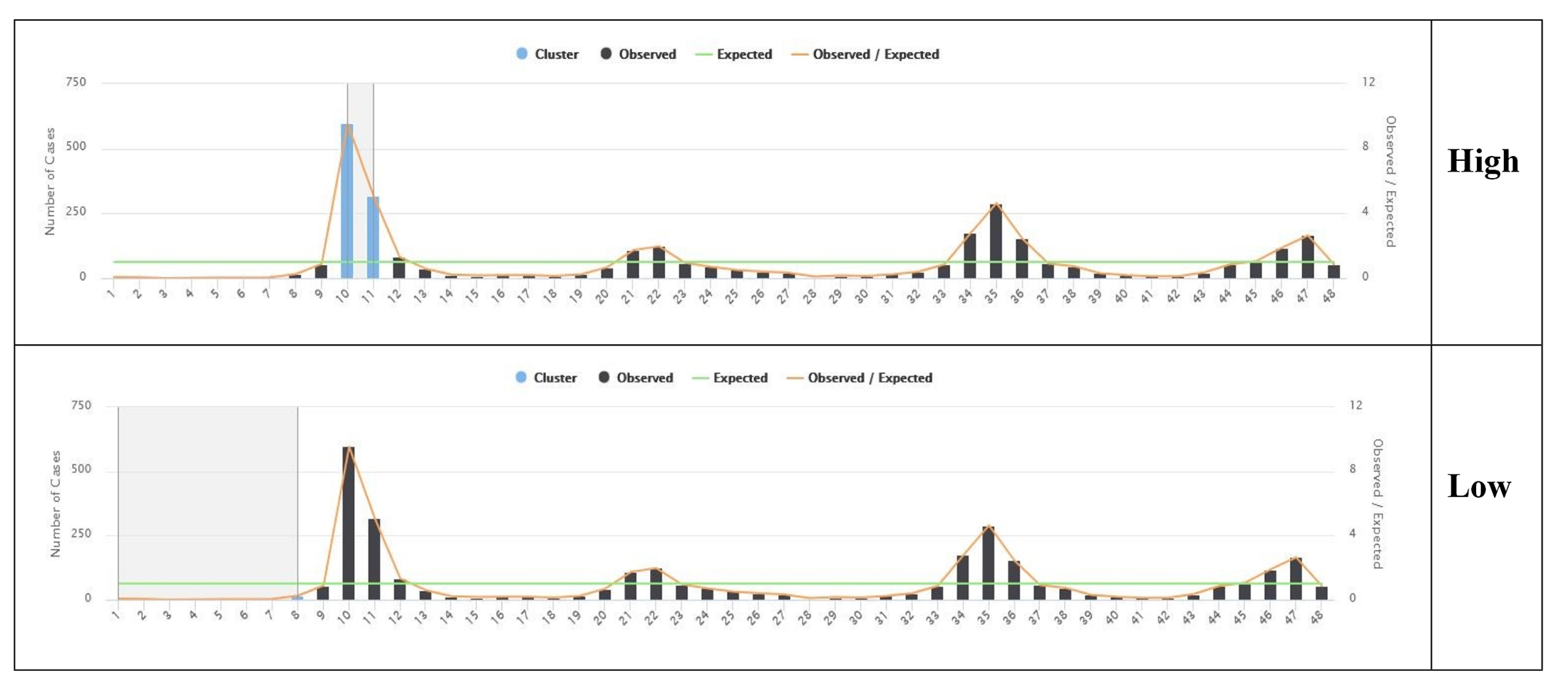

Supplement: S2 Fig — (TIF) [file pone.0320990.s004.tif]
