# Supplementary material for: National spatiotemporal patterns of influenza-like illness in Iran: A pre-COVID-19 study (2015–2019)
Source: PLoS One. 2025 Apr 21;20(4):e0320990. doi: 10.1371/journal.pone.0320990 (PMC12011232; doi:10.1371/journal.pone.0320990)
Supplement: Attachment — Submitted filename: Response to Reviewers .docx [file pone.0320990.s006.docx]

**Response to Reviewers**

**Dear editor,**

We are grateful for your consideration of this manuscript, and we very much appreciate your suggestions, which have been very helpful in improving the manuscript. We also thank the reviewers for their careful reading of our text and providing invaluable comments.

All the comments we received on this study have been taken into account and addressed or responded. In this letter, we present our reply to each of them separately.

Two versions of the manuscript are enclosed, one where all the changes have been track-changed, and another version without any marks.

We hope that these changes to the manuscript will facilitate the decision to publish this study in your journal. We are open to consideration of any further comment on our answers.

**Sincerely,**

**The authors**

**Journal Requirements:**

1. Please ensure that your manuscript meets PLOS ONE's style requirements, including those for file naming.

**Authors’ answer:** Thank you for providing your feedback and guidance regarding our manuscript. We have reviewed the journal's style requirements and ensured that the manuscript complies fully with PLOS ONE's formatting and style guidelines. Additionally, all file names have been updated to meet the specified criteria.

2. PLOS requires an ORCID iD for the corresponding author in Editorial Manager on papers submitted after December 6th, 2016. Please ensure that you have an ORCID iD and that it is validated in Editorial Manager. To do this, go to ‘Update my Information’ (in the upper left-hand corner of the main menu), and click on the Fetch/Validate link next to the ORCID field. This will take you to the ORCID site and allow you to create a new iD or authenticate a pre-existing iD in Editorial Manager.

**Authors’ answer:** Thank you for the reminder regarding the requirement for an ORCID for the corresponding author. I confirm that I have completed this step and successfully validated my ORCID in the Editorial Manager system.

3. We note that Figures 2-4 & S1 in your submission contain [map/satellite] images which may be copyrighted. All PLOS content is published under the Creative Commons Attribution License (CC BY 4.0), which means that the manuscript, images, and Supporting Information files will be freely available online, and any third party is permitted to access, download, copy, distribute, and use these materials in any way, even commercially, with proper attribution. For these reasons, we cannot publish previously copyrighted maps or satellite images created using proprietary data, such as Google software (Google Maps, Street View, and Earth).

**Authors’ answer:** Thank you for bringing this to our attention. To ensure compliance with PLOS ONE's copyright policy, we have removed the satellite layers from the maps in Figures 2–4 and S1. The updated figures now utilize openly available data and sources that align with the Creative Commons Attribution License (CC BY 4.0).

**Editor comment:**
A clear presentation of case definition (either clinical or lab-based) and correct interpretation of results based on the type of samples you have used is mandatory for acceptance of the paper.

**Authors’ answer:**

Thank you for this comment. After consulting with the data administrator, we confirmed that the diagnostic test was conducted solely to differentiate between influenza and non-influenza cases. It was not performed for all patients but only for those for whom the doctor suspected of having influenza, a notifiable disease, and deemed testing necessary. Laboratory testing, using real-time reverse transcription-polymerase chain reaction (RT-PCR) assays, was exclusively applied to influenza. For positive cases, the specific pathogen type (A-type [H1N1, H3N2] or B-type) was recorded, and we categorized our results accordingly.

For patients who were not tested or received a negative test result, doctors relied on clinical assessment to make an inferential diagnosis, which we had originally mentioned in the paper. However, since this approach may be subject to bias and is not directly relevant to our primary aim of analyzing the spatiotemporal patterns of influenza-like illnesses (ILI), we decided to categorize these cases as “other respiratory diseases,” in alignment with Reviewer 2’s comment. The maps and most of the paper remain unchanged, with modifications made only to Table 1, specifically in the diagnosis section.

We also made some changes to the methods section to clarify this (Page 5, Lines 106-111):
*“The diagnostic test was conducted solely to differentiate between influenza and non-influenza cases and was performed only for patients suspected of having influenza, a notifiable disease. Real-time reverse transcription-polymerase chain reaction (RT-PCR) assays were used exclusively for influenza detection, with positive cases further classified by pathogen type (A-type [H1N1, H3N2] or B-type). As a result, the diagnoses in this study have been classified into two categories: influenza and other respiratory infections.”*

**Reviewers' comments:**

**Reviewer #1:**

The authors reported the spatiotemporal patterns of influenza-like illness in Iran. They appropriately discussed the regional differences in the epidemic situation and the reasons for these differences. I found this to be a highly significant study.
I have two questions for the authors.

**Authors’ answer:** Thank you for taking the time to review our manuscript. We have addressed all your comments as follows.

1. In Table 1, the test results are described, and about half of the results were negative. What kind of tests were used for these results? For example, even for rapid influenza virus tests, I think the sensitivity is too low.

**Authors’ answer:**

We thank you for your insightful comments. As we mentioned in our response to the editor, laboratory testing using RT-PCR was conducted exclusively to confirm influenza cases, and no rapid influenza diagnostic tests were used. Therefore, many patients with influenza-like illness (ILI) tested negative for influenza, as their symptoms were caused by other respiratory infections. The use of RT-PCR to diagnose influenza in Iran was also previously reported in another study [1].

2. Additionally, regarding the types of influenza viruses, nearly 90% are categorized as "other" rather than type A or B. Please provide a breakdown of the "other" category.

**Authors’ answer:** Thank you for identifying this error in our calculation. Upon reviewing the table, we found that the counts for influenza types A and B included all records rather than being restricted to positive cases. We have now corrected this by including only positive cases in the calculations, resulting in updated values in the relevant table.

Additionally, the category previously labeled as "other" has been revised to "not reported" to more accurately reflect the nature of the data. These revisions have been incorporated into both the manuscript and the updated table. We trust that these adjustments adequately address your concerns (Page 7, Table 1: virus type).

“

| Virus type:  A   - H1N1 - H3N2 - H9 - Other subtyped - Un-subtyped - Not reported   B  Not reported | 4,601 (79.77)  1,792 (38.95)  2,138 (46.47)  0 (0.00)  2 (0.04)  255 (5.54)  414 (9)  1,126 (19.52)  41 (0.71) | 5,517 (78.63)  2,041 (36.99)  2,687 (48.70)  1 (0.02)  3 (0.05)  305 (5.53)  480 (8.71)  1,462 (20.83)  37 (0.53) | 554 (86.56)  309 (55.78)  161 (29.06)  0 (0.00)  0 (0.00)  33 (5.96)  51 (9.20)  78 (12.19)  8 (1.25) | 9,564 (78.75)  3,524 (36.84)  4,664 (48.77)  1 (0.01)  5 (0.05)  527 (5.51)  843 (8.82)  2,510 (20.67)  70 (0.58) | 10,118 (79.15)  3,833 (37.88)  4,825 (47.69)  1 (0.01)  5 (0.05)  560 (5.53)  894 (8.84)  2,588 (20.24)  78 (0.61) |
| --- | --- | --- | --- | --- | --- |

“

**Reviewer #2:**

The manuscript "National Spatiotemporal Patterns of Influenza-Like Illness in Iran: A Pre-COVID-19 Study (2015–2019)" explores the geographic and temporal trends of influenza-like illness in Iran, providing valuable insights into pre-pandemic disease dynamics and public health preparedness.
The manuscript is well written; however, I have the following concerns.
(a) Under the sub-title methods, the authors stated in Lines 114 and 115 that Diagnoses in this study were determined through differential diagnosis based on clinical symptoms and assessments. No laboratory tests were performed to confirm these diagnoses.

1. Clarification of Methods needed:
• The authors should provide explicit details on how the listed microorganisms or infections were diagnosed without laboratory testing. If laboratory assays were conducted, the methods should be amended to reflect this, including the protocols used.

**Authors’ answer:** We appreciate your thoughtful feedback on the methods section. To address your concerns and provide greater clarity, we would like to offer the following explanation:

As outlined in **Table 1**, laboratory testing was performed exclusively to confirm influenza cases among respiratory infections. The proportion of notifications with laboratory-confirmed influenza is clearly presented in the table. For all other respiratory conditions, no laboratory tests were conducted. Instead, diagnoses were determined through clinical assessments and differential diagnosis, based on patients’ symptoms and the prevailing epidemiological context.

To ensure complete transparency and resolve any potential ambiguities, we have revised the methods section to explicitly clarify that laboratory testing was limited to influenza diagnosis, while other respiratory conditions were identified solely through clinical evaluation.

We trust this clarification addresses your concerns and enhances the overall clarity and precision of the manuscript (Page 5, Lines 106-111).

*“The diagnostic test was conducted solely to differentiate between influenza and non-influenza cases and was performed only for patients suspected of having influenza, a notifiable disease. Real-time reverse transcription-polymerase chain reaction (RT-PCR) assays were used exclusively for influenza detection, with positive cases further classified by pathogen type (A-type [H1N1, H3N2] or B-type). As a result, the diagnoses in this study have been classified into two categories: influenza and other respiratory infections.”*

2. Revision of Results Section:
• The authors should justify the inclusion of specific infections and sample types in the results, correlating these with the stated methodology. If certain infections were inferred based on clinical criteria, the diagnostic criteria for each should be detailed.

**Authors’ answer:** We thank the reviewer for the insightful feedback. The differential diagnosis of respiratory conditions in this study was based on the documented clinical history and symptomatology of patients, without utilizing laboratory assays. Laboratory testing was exclusively employed for the confirmation of influenza cases, and the results of these diagnostic tests, aligned with the sample types detailed in the Results section, are presented in Table 1. To ensure the scientific rigor and credibility of our findings, we have revised the terminology used in the Results section. Specific infections initially mentioned have been consolidated under the broader term "other respiratory infections," as no laboratory assays were performed to confirm these diagnoses. Influenza, being the sole condition for which laboratory confirmation was conducted, remains explicitly stated in both the text and Table 1. These revisions have been incorporated into the manuscript to align the Results section more closely with the study's methodology and the scope of diagnostic testing employed (Page 7, Table 1: Diagnosis)

“

| Diagnosis:  Influenza  Other respiratory infections | 5,768 (11.14)  45,987 (88.86) | 7,016 (12.06)  51,148 (87.94) | 640 (20.46)  2,488 (79.54) | 12,144 (11.37)  94,647 (88.63) | 12,784 (11.63)  97,135 (88.37) |
| --- | --- | --- | --- | --- | --- |

“

3. Limitations:
• The limitations of relying solely on clinical diagnoses should be transparently discussed. The authors should highlight the potential for diagnostic inaccuracies and acknowledge the absence of laboratory confirmation.

**Authors’ answer:** We thank the reviewer for this valuable comment. The lack of laboratory confirmation for non-influenza respiratory conditions has been acknowledged as a potential limitation of this study. However, given that the primary focus was to examine the spatiotemporal patterns of influenza-like illness across Iran, this limitation is unlikely to have introduced significant bias or affected the overall findings. This point has been explicitly addressed in the revised "Limitations" section to ensure clarity and transparency regarding the scope and implications of our study (Page 16, Lines 328-331).

*“Third, the lack of laboratory confirmation for non-influenza respiratory conditions could be regarded as a limitation of this study. Nevertheless, as the primary objective was to analyze the spatiotemporal patterns of ILI at the county level, this limitation is unlikely to have had a significant impact on our aim. “*

4. Consider Alternative Approaches:
• If laboratory assays were not done, the authors should limit their claims to broad categories of diseases (e.g., "respiratory infections") rather than naming specific pathogens.

**Authors’ answer:** We thank the reviewers for insightful comment and fully agree with your observation regarding the specificity of pathogen identification in the manuscript. To address this concern and enhance clarity, we have revised the diagnostic classifications used in the study.

We have updated the diagnostic categories to two broad classifications: influenza and other respiratory infections. This revision ensures that the claims made in the manuscript accurately reflect the study methodology.

These changes have been incorporated into the Results section, with corresponding updates made to the relevant table. This adjustment clarifies that specific pathogen identification, beyond influenza, was not performed, in line with the diagnostic approach employed.

We trust that this revision effectively addresses your concern and enhances the transparency and accuracy of the manuscript (Page 7, Table 1: Diagnosis).

“

| Diagnosis:  Influenza  Other respiratory infections | 5,768 (11.14)  45,987 (88.86) | 7,016 (12.06)  51,148 (87.94) | 640 (20.46)  2,488 (79.54) | 12,144 (11.37)  94,647 (88.63) | 12,784 (11.63)  97,135 (88.37) |
| --- | --- | --- | --- | --- | --- |

“

(Abstract, Lines: 22 to 25)

*“Among all patients, 53% were tested for influenza, and 22% of those tested had a confirmed case, which accounts for 12% of all ILI patients, with Type A being the predominant strain, accounting for 79.15% of cases. Type A influenza had a higher CFR than type B (5.4% vs. 3.01%, respectively). Among the A virus subtypes, H1N1 exhibited the highest CFR at 8.06%.”*

(Page: 6, Lines: 141-146)

*“Among all patients, 53% were tested for influenza, and 22% of those tested had a confirmed case, which accounts for 12% of all ILI patients (n=12,784). Of the type of influenza virus (A and B), type A was the most contagious, accounting for 79.15% cases compared to 20.24% for type B. Additionally, type A had a higher case fatality rate (CFR) than type B (5.4% vs. 3.01%, respectively). With the A virus subtypes, H1N1 had the highest CFR at 8.06%, while H3N2 was responsible for the highest percentage of infections (n=4,825, 47.69%).”*

(b) The influence of environmental and socio-economic factors is mentioned but not integrated into the analysis explicitly. The authors should consider incorporating covariates like climate variables (e.g., temperature, and humidity) and socio-economic indicators (e.g., healthcare access) into the spatiotemporal model.

**Authors’ answer:** We sincerely appreciate your comment regarding the inclusion of environmental and socio-economic factors in our analysis. We would like to provide further clarification on the focus and scope of our study in relation to these factors.

The primary objective of our study was to explore the spatiotemporal patterns of influenza-like illness (ILI) in Iran. Although we have mentioned the potential impacts of environmental factors (such as temperature and humidity) and socio-economic indicators (such as healthcare access) on disease patterns, these variables were not directly incorporated into our analysis.

Any mention of these factors in the Discussion section was intended to highlight potential avenues for future research, rather than to draw definitive conclusions based on the current study.

To enhance the clarity of the manuscript, we have revised the text to more explicitly state that the examination of environmental and socio-economic factors is beyond the scope of the present study and should be considered as a hypothesis for further exploration. We believe this revision will provide a clearer focus on the objectives of our study while suggesting directions for future research (Page 14, Lines: 274-275).

“Given these assumptions, future studies might consider incorporating covariates such as climatic factors and socioeconomic indices into spatiotemporal models.“

(c) Table I has Men (%) and Women (%) as categories. My concern is that are the children under 5 years also classified as men and women. How can this be married with the gender classification at the end of the table as male and female? These make the table very difficult to comprehend.

**Authors’ answer:** Thank you for your helpful comment regarding the categorization in Table 1. To avoid any confusion and to ensure clarity, we have revised the table to use "Male" and "Female" consistently across all age groups, including children under 5 years old. This change should improve the readability and comprehensibility of the table (Page 7, Table 1, line: 1).

“

|  | Male (%) | Female (%) | Death (%) | Recovery (%) | Total (%) |
| --- | --- | --- | --- | --- | --- |

“

**References:**

1.	MohammadEbrahimi S, Kiani B, Rahmatinejad Z, Baral S, Hashtarkhani S, Dehghan-Tezerjani M, et al. Geospatial epidemiology of hospitalized patients with a positive influenza assay: A nationwide study in Iran, 2016–2018. PLOS ONE. 2022;17: e0278900. doi:10.1371/journal.pone.0278900
